# Nanostructured Perovskite Solar Cells

**DOI:** 10.3390/nano9101481

**Published:** 2019-10-18

**Authors:** Calum McDonald, Chengsheng Ni, Paul Maguire, Paul Connor, John T. S. Irvine, Davide Mariotti, Vladimir Svrcek

**Affiliations:** 1 Research Center for Photovoltaics, National Institute of Advanced Industrial Science and Technology (AIST), Tsukuba, Ibaraki 305-8568, Japan; vladimir.svrcek@aist.go.jp; 2 College of Resources and Environment, Southwest University, Beibei, Chongqing 400715, China; nichengsheg@163.com; 3 School of Engineering, Ulster University, Newtownabbey BT37 0QB, UK; pd.maguire@ulster.ac.uk (P.M.); d.mariotti@ulster.ac.uk (D.M.); 4 School of Chemistry, University of St Andrews, North Haugh, St Andrews KY16 9AJ, UK; pac5@st-andrews.ac.uk (P.C.); jtsi@st-andrews.ac.uk (J.T.S.I.)

**Keywords:** solar cells, perovskites, perovskite nanocrystals, perovskite quantum dots, low-dimensional perovskites, nanocrystal solar cells, organic–inorganic hybrid solar cells, lead halide solar cells, hybrid solar cells

## Abstract

Over the past decade, lead halide perovskites have emerged as one of the leading photovoltaic materials due to their long carrier lifetimes, high absorption coefficients, high tolerance to defects, and facile processing methods. With a bandgap of ~1.6 eV, lead halide perovskite solar cells have achieved power conversion efficiencies in excess of 25%. Despite this, poor material stability along with lead contamination remains a significant barrier to commercialization. Recently, low-dimensional perovskites, where at least one of the structural dimensions is measured on the nanoscale, have demonstrated significantly higher stabilities, and although their power conversion efficiencies are slightly lower, these materials also open up the possibility of quantum-confinement effects such as carrier multiplication. Furthermore, both bulk perovskites and low-dimensional perovskites have been demonstrated to form hybrids with silicon nanocrystals, where numerous device architectures can be exploited to improve efficiency. In this review, we provide an overview of perovskite solar cells, and report the current progress in nanoscale perovskites, such as low-dimensional perovskites, perovskite quantum dots, and perovskite-nanocrystal hybrid solar cells.

## 1. Introduction

In the search of high-efficiency, low-cost solar cells, a multitude of new materials and architectures are currently being explored. Over the past decade, organometal halide perovskites (OHPs) have emerged as a highly promising photovoltaic material and have been demonstrated as the active layer in perovskite solar cells (PSCs) with efficiencies over 25% for laboratory-based devices (~0.1 cm^2^) [1] and around 10–15% in modules [2] and are recently being employed in high-efficiency tandem devices [3]. The performance of PSCs has seen a meteoric rise over the past decade and they are already comparable with or superior to well-established photovoltaic technologies [1]. OHPs are attractive particularly due to their ease of processing [4], large absorption coefficients [5], long carrier diffusion lengths [6], low exciton binding energies [7], and low non-radiative recombination rates [8]. These properties also make OHPs an attractive material for various other optoelectronic devices, such as light emitting diodes [9], lasers [10,11], and photodetectors [12].

**Figure 1:**
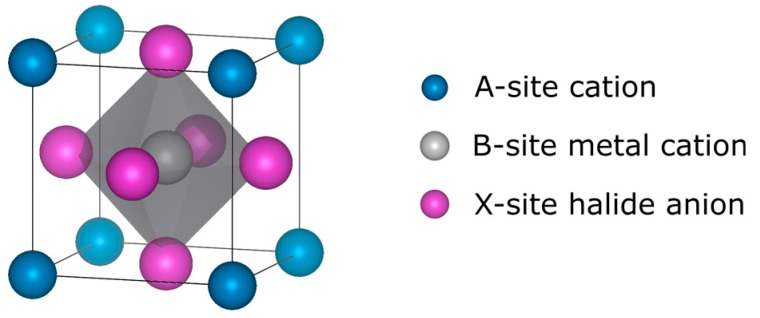
Cubic perovskite unit cell.

OHPs have a perovskite crystal structure with the general stoichiometry ABX_3_ as shown in Figure 1. The A-site is occupied by a monovalent cation e.g., methylammonium (MA, CH_3_NH_3_^+^), formamidinium (FA, CH_3_(NH_2_)_2_^+^), Cs^+^ etc. The B-site is usually occupied by a Pb^2+^ divalent metal cation and can be substituted by a similarly-sized divalent cation such as Sn^2+^. The X-site is usually occupied by a halide anion e.g., I^−^, Cl^−^, Br^−^. OHPs with mixed cations and/or anions are now the standard for high efficiency cells, particularly due to improved structural stability [13,14,15]. Their high compositional tunability, whereby the bandgap can be easily modified through ion substitution [16] and low-cost facile deposition procedures [17] makes OHPs excellent candidates for tandem solar cells, where two materials of different bandgaps are employed in conjunction to absorb different parts of the solar spectrum. OHPs can be employed either as the top cell in a tandem device (with e.g., silicon, cadmium telluride, copper indium gallium diselenide etc. bottom cell) or in a stacked perovskite–perovskite tandem device. The successful fabrication of tandem cells with OHPs has the potential to achieve efficiencies in excess of 40% [3].

While OHPs have demonstrated remarkable efficiencies in laboratory solar cells, there remains significant challenges regarding long-term suitability and feasibility of commercialization [18]. OHPs are extremely susceptible to moisture-induced degradation, and therefore devices must be fabricated in controlled nitrogen atmospheres to avoid trapped moisture in the active layer. Furthermore, devices must be sufficiently encapsulated to prevent external moisture ingress, and the fragility of OHPs along with weak inter-layer adhesion may demand rigid glass substrates to avoid delamination or fractures in the OHP. Even so, heat and light cycling can still induce degradation in encapsulated devices due to thermal mismatch [19]. The use of encapsulants, which can be expensive, along with rigid glass supports, makes OHPs less attractive due to increased costs [3]. It is therefore highly desirable to develop perovskite materials which are stable and tolerant to moisture and other environmental stresses.

Forming nanostructured OHPs (also referred to as low-dimensional OHPs) can be a potential route towards increasing the stability. So far, various types of low-dimensional OHPs have been demonstrated in solar cells, and typically show far superior stability to bulk OHPs [20,21,22]. This is achieved particularly due to higher formation energies of the low-dimensional perovskite structure and the possibility of encapsulating low-dimensional OHPs in long-chain polymers, essentially providing a protective barrier to moisture [22]. However, carrier transport tends to be restricted in nanostructured perovskites due to the presence of potential barriers within the nanostructured OHP, while quantum confinement also tends to widen the bandgap towards values typically in excess of 2 eV. This therefore comes at a cost to the performance, with the best nanostructured OHPs performing between 10–18% [20,21,22,23,24]. 

Considering the recent advances in nanostructured perovskites, here we will provide an insight into the important developments and progress in photovoltaics. First, an introduction to the use of bulk OHPs in solar cells will be provided while discussing the challenges and issues facing these materials in order to provide a context for the recent direction towards nanostructured perovskites. This review will then provide a perspective into nanostructured perovskite solar cells as a possible route towards overcoming the issues pertaining to bulk OHPs. Furthermore, hybrid devices formed with OHPs and nanocrystals (NCs) will be discussed, along with high-stability metal oxide perovskite nanocrystals. We hope this will provide the reader with a basis for understanding the current status of PSCs and the potential opportunities of stable, low-dimensional perovskites.

## 2. Overview of Bulk Perovskite Solar Cells

PSCs were initially inspired by the dye-sensitized solar cell (DSSC), where simply replacing the dye in a DSSC with an OHP immediately yielded efficiencies of ~3% [25]. The OHPs used were either MAPbI_3_ or MAPbBr_3_, where MA is the small organic cation methylammonium (CH_3_NH_3_^+^). Since the liquid electrolyte, which is used in DSSCs as a redox mediator, dissolved the OHP, these devices had very short lifetimes on the order of seconds. The rapid dissolution of the OHP was overcome by replacing the liquid electrolyte with a polymer which did not dissolve the OHP. Subsequently, devices were reported using the polymer spiro-MeOTAD for hole transport, quickly achieving efficiencies of ~10% with improved device lifetime [26,27]. It was demonstrated that electron and hole transport occurs in the OHP, indicating that free-carriers are generated in the OHP with long diffusion lengths and lifetimes, contrary to suspicion that photocarriers would be excitonic as for organic solar cells, and therefore the sensitized architecture was in fact not necessary [26].

**Figure 2:**
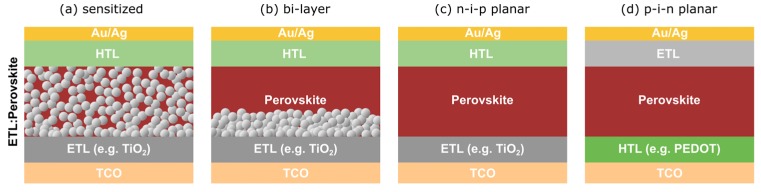
Various device architectures for organometal trihalide perovskite solar cells. (**a**) Mesoporous sensitized, (**b**) bi-layer, (**c**) n-i-p planar and (**d**) p-i-n planar. ETL, HTL, and TCO stand for electron transport layer, hole transport layer, and transparent conducting oxide, respectively.

The main PSC device architectures are shown in Figure 2. The OHP is sandwiched between two selective contacts, an electron transport layer (ETL) such as TiO_2_, and a hole transport layer (HTL) such as spiro-OMeTAD. Metallic contacts are formed on either side of the transport layers: a window contact is formed using a transparent conducting oxide (TCO) such as indium-doped tin oxide (ITO), and a back contact is formed using either gold, silver, aluminum etc. The first architecture employed in the research timeline was the sensitized architecture using a thick mesoporous layer of TiO_2_ (Figure 2a). This was quickly replaced with bi-layer devices, where the mesoporous-TiO_2_ was reduced in thickness and a thicker OHP layer was deposited to allow for greater absorption of light and longer crystalline order with larger grain sizes (Figure 2b). A planar device architecture can also be used, with either n-i-p configuration (Figure 2c) or p-i-n configuration (Figure 2d). The planar device eliminates the necessity for the mesoporous TiO_2_ layer, further reducing fabrication costs and complexity. Planar devices show greater potential for low-cost roll-to-roll printing of PSCs at low temperatures due to the elimination of mesoporous-TiO_2_ which must typically be annealed at high temperatures during device fabrication (~500 °C) for high-efficiency PCSs, and is therefore unattractive for large-scale production while also eliminating the possibility of fabricating devices on flexible plastic substrates. Furthermore, the high-temperature annealing of TiO_2_ is not suitable for the fabrication of tandem devices with silicon or perovskite bottom cells since such high-temperature annealing process will damage the silicon bottom cell [3]. Planar devices using an SnO_2_ electron transport layer can be fabricated via low-temperature methods and demonstrate superior stability to mesoporous-TiO_2_ devices, however the best efficiency of 21.6% is somewhat lower than mesoporous-TiO_2_ devices (25.2%) [1,28]. Since PSCs employing mesoporous-TiO_2_ transport layers have shown greater efficiencies than planar devices thus far [29], ideally low-temperature fabrication techniques should be developed for mesoporous-TiO_2_ transport layers to enable their incorporation into tandem devices. 

### 2.1. Stability of Perovskite Solar Cells

**Figure 3:**
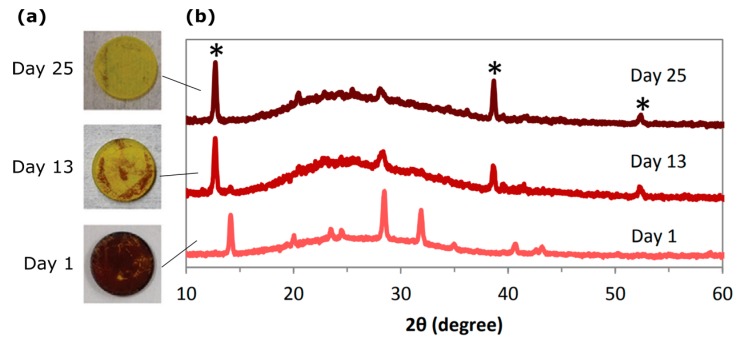
Degradation of MAPbI_3_. (**a**) Photographs of MAPbI_3_ degradation and (**b**) corresponding X-ray diffraction (XRD) spectra of the same samples after 1, 13, and 26 days stored in ambient conditions. The starred peaks in the XRD spectra correspond to PbI_2_. Reproduced from ref. [30], with permission from John Wiley and Sons, 2016.

While exceptional efficiencies have been demonstrated with Pb-based perovskites [13,14,15], significant challenges exist such as poor stability, toxicity, and rate-dependent current-voltage hysteresis. Stability is an important consideration when assessing commercialization viability of new materials given that silicon solar cells can easily operate for >25 years, even when exposed to a broad range of temperatures and intense solar irradiance. OHPs tend to degrade rapidly in open air conditions and must be fabricated in controlled atmospheres to avoid moisture contamination. The rapid degradation of MAPbI_3_ in open-air conditions is shown in Figure 3, where the majority of the MAPbI_3_ layer degraded to PbI_2_ within 13 days [30]. Although the exact mechanism of degradation remains unclear; it is generally understood that an intermediate phase is first formed via hydration of the OHP [31,32]. Considering the decomposition of MAPbI_3_, the hydration of MAPbI_3_ leads to its conversion to MA_4_PbI_6_·2H_2_O and PbI_2_, followed by phase separation and the subsequent loss of MA, with the final products being CH_3_NH_3_I, PbI_2_, and H_2_O [31]. The degradation has been shown first to occur at the grain boundaries and is assisted by the presence of trapped charges which usually exist at defect sites, surfaces, and grain boundaries [33]. Ions can easily migrate within OHPs, causing charge accumulation, phase segregation, lattice distortions, and strain in the perovskite structure [34,35,36,37,38]. The degradation of OHPs is enhanced under illumination, and degradation can be accelerated even under moderate temperatures of ~60 °C [39,40]. Furthermore, I_2_, which is generated within the OHP due to exposure to moisture, can easily migrate and leads to the self-sustaining and irreversible degradation of the OHP [41]. The degradation of OHPs leads to the release of the gaseous products CH_3_NH_2_, HX, CH_3_X, and NH_3_ (where X is a halide), and the release of these gases can be observed at temperatures below 70 °C [42].

Due to the high susceptibility of OHPs to degrade when exposed to moisture, it is therefore necessary to carefully control the atmosphere during fabrication. Entire device encapsulation is necessary to prevent exposure to moisture and mechanical fractures. For encapsulated devices, the formation of bubbles has been observed in the encapsulant layer due to the release of gaseous species. Encapsulation prevents gaseous products from escaping, creating a thermodynamically enclosed system which is expected to reduce the rate of degradation [42]. Encapsulation is therefore essential for several reasons: to prevent the ingress of moisture; to prevent the release of gases; and to prevent the release of toxic materials to the environment. However, due to the thermal expansion coefficient mismatch between the various layers, including the encapsulant, temperature cycling of the PSC (i.e., day and night temperature variations) can lead to significant delamination and device failure. Careful selection of the encapsulant and various device layers is therefore necessary to minimize delamination caused by temperature cycling. This eliminates the possibility of flexible, low-weight modules, and the low stability and Pb-contamination necessitates careful recycling of PSCs. In spite of these measures, the question of whether the lifetime of OHPs can match silicon PV remains dubious.

### 2.2. Toxicity of Perovskite Solar Cells

Pb-containing OHPs’ decomposition results in the formation of Pb-halide compounds, metallic Pb, and various carbonated molecules [43]. Although PSCs contain small amounts of Pb (~0.4 g/m^2^ for a 400 µm-thick OHP layer) [44], the harmful Pb-halides generated via degradation are highly water-soluble and therefore pose a significant risk to the environment [45]. The contamination of Pb can be addressed either by replacing Pb with other non-toxic elements or by stabilizing the structure of the perovskite so as to avoid the formation of PbI_2_. Unfortunately, computational studies have suggested that there is no viable alternative to Pb in PSCs to achieve the similarly high efficiencies which are in excess of 20% [46]. The high efficiencies of OHPs is attributed to the favorable Pb^2+^ orbital hybridization with I^-^ and Br^-^ halide ions which results in high absorption coefficients and long carrier diffusion lengths [47]. Sn is a potential alternative to Pb, and whilst still toxic to animals and humans, it is less harmful than Pb. [43] Sn-OHPs have been produced by the direct replacement of Pb with Sn, but the best efficiency achieved to date is 7.14% [23]. In addition, the stability of Sn-based devices is usually worse than Pb-OHPs due to the tendency of tin to easily oxidize from Sn^2+^ to Sn^4+^. This can be mitigated to some extent by the addition of SnF_2_ and ethylenediammonium during fabrication to inhibit the formation of Sn^4+^ [23,48]. While pure Sn-OHPs are unstable, the oxidation of Sn^2+^ becomes less energetically favorable when less than 50% of the B-site in the perovskite structure is occupied by Sn^2+^ (i.e., MAPb_≥0.5_Sn_≤0.5_I_3_) and the stability is significantly improved [49]. Notably, Zn, which is a 2+ ion with a slightly smaller ionic radius than Pb, has also been investigated for the partial replacement of Pb and has demonstrated an improvement in the power conversion efficiency (PCE) for small amounts of Zn (~1% to 5%). The introduction of Zn into MAPbI_3_ leads to the formation of larger grains which are more homogeneous, and layers which are more compact and with fewer pinholes. This is achieved through a lattice contraction induced by the smaller Zn ion, along with stronger coordination with the organic cation, leading to a reduction in the amount of point defects [50,51,52,53]. However, this work only serves to reduce Pb contamination without eliminating it entirely, and the contamination of toxic Pb and Sn remains and degradation is still observed [49].

### 2.3. Hysteresis in PSCs

**Figure 4:**
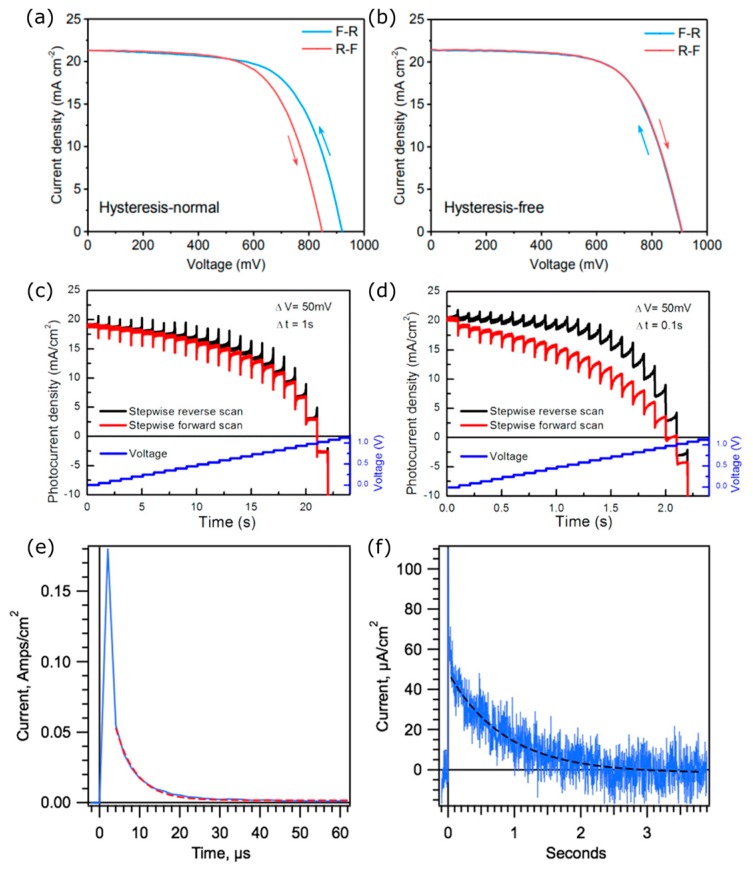
(**a,b**) Current density-voltage curves with forward (R-F) and reverse (F-R) voltage scan direction for a device with hysteresis (**a**) and without (**b**). Reproduced from ref. [66], with permission from The Royal Society of Chemistry, 2017. (**c**,**d**) Time-dependent photocurrent response under reverse and forward stepped scans with (b) 1 s step time and (c) 0.1 s step time. Reproduced from ref. [67], with permission from American Chemical Society, 2015. (**e**,**f**) Current decay after removing device from illumination showing two discharging events occurring over different timescales. Reproduced from ref. [68], with permission from American Chemical Society, 2015.

A common issue exhibited by nearly all PSCs is a hysteresis present during solar cell characterization. Hysteresis, defined as the dependence of the state of a system on its history, is frequently observed during current density-voltage (J-V) measurements, where a change in the voltage scan direction between forward and backward results in a differing J-V response, as shown in Figure 4a. A device without J-V hysteresis is shown in Figure 4b. The observed hysteresis is largely attributed to ion mobility within the OHP [54,55,56], whilst other mechanisms have also been proposed, see reference [57]. Hysteresis is problematic as it primarily introduces difficulties in accurately measuring device performance, but can also be indicative of stability issues [41,58]. Recent work [13,15] has shown that high-efficiency mesoscopic devices possess low hysteresis in the forward and backward J-V scans with the same scan rates from 10 mV/s to 50 mV/s; however, hysteresis is still well observed particularly for fast scans [56,59,60]. Selecting appropriate contacts and forming high-quality OHP layers appears to negate most of the hysteresis observed during standard performance measurements with slow scan speeds; however, the J-V character for fast scans is often unreported and ionic motion and charge accumulation are still likely to be present in the perovskite layer. Furthermore, hysteresis is often intensified as devices are scaled to active areas over 1 cm^2^, particularly due to issues with controlling morphology when depositing OHPs over larger areas [61]. The hysteresis observed in OHPs depends on various measurement conditions during the J-V characterization, in particular: the voltage scan rate and scan range [56,62]; the delay time between applying the bias voltage and measuring the current [63]; and the poling voltage prior to measurement [57]. Hysteresis has also been shown to vary with the grain size of the perovskite [57,64], the A-site cation [65], and device architecture [62,63].

The hysteresis is well-described by Figure 4c,d whereby the voltage is scanned forward and backward in a stepwise fashion with different delay times between the steps: 1 s in Figure 4c and 0.1 s in Figure 4d [67]. It is clear that at least two processes are involved: one is an ultrafast process which leads to an almost instantaneous (microsecond) change in photocurrent, followed by a slower response on the timescale of milliseconds to seconds. There is a large difference in the forward and reverse J-V scans observed for a 0.1 s voltage step time: this arises because when the step speed is too fast, the photocurrent is not able to stabilize and there is a remnant charge stored in the device. This was further investigated and it was shown that there are at least two ways in which charge is stored in OHPs (Figure 4e,f) [68]. After removing an OHP device from illumination, the photogenerated current decayed from 180 mA/cm^2^ to less than 50 μA/cm^2^ within 50 μs (Figure 4e). This was followed by a second, longer decay event which occurred over the next ~3 s (Figure 4f). Although the peak current in the second decay event (~50 μA/cm^2^) accounted for less than 1% of the initial photocurrent (~180 mA/cm^2^), the lifetime of the second current was far longer and therefore the total charge associated with this slower decay was calculated to be ~50 times larger than the charge associated with the initial microsecond-discharge event. Therefore, at least two types of capacitive electronic charges were confirmed in OHPs: the first one is small (~0.2 μC cm^−2^) and likely due to charge trapping; and the second one is much larger (~40 μC cm^−2^), which could be the result of mobile ions or dipole realignment [68]. Furthermore, it is also known that large differences in the carrier mobility of the electron and hole transport layers can lead to charge accumulation resulting in hysteresis [68].

**Figure 5:**
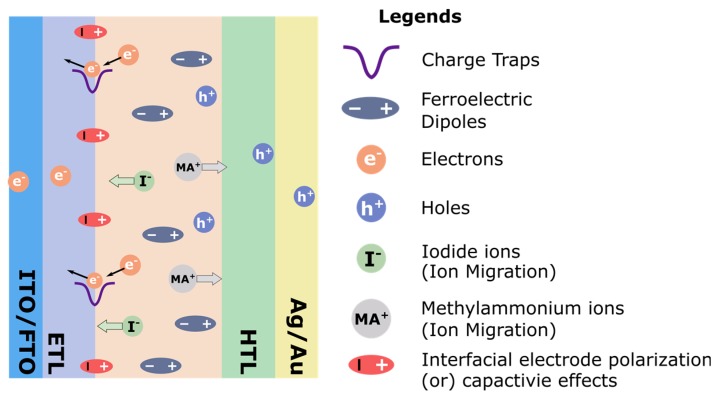
Schematic of the proposed contributions to hysteresis. ITO, FTO, ETL and HTL stand for indium-doped tin oxide, fluorine-doped tin oxide, electron transport layer, and hole transport layer, respectively. Reproduced from ref. [76], with permission from Elsevier, 2016.

Understanding the origin and mechanism of hysteresis could lead to the improvement of the performance and stability of PSCs. The main mechanisms which have been proposed to contribute to the effect are: ion migration [56,67,69], charge trapping and accumulation [70,71], and polarization of dipoles [57,62,72]. These mechanisms are represented in Figure 5 and are described briefly in order of the legend:

1. Charge traps: Charges can become trapped at defects on surfaces or at grain boundaries and induce recombination, reducing the photocurrent.
2. Ferroelectric dipoles: Some reports have indicated that OHPs such as MAPbI_3_ are ferroelectric, and the polarization of domains would modify carrier transport through the perovskite, resulting in the observed hysteresis [62,73,74,75].
3. Electrons and holes: Similar to charge trapping, electrons and holes can accumulate in transport layers due to defects or imbalances in the carrier mobilities of the electron and hole transport layers.
4. Ion migration: Iodide ions and methylammonium ions can migrate to interfaces under applied bias and alter the internal field reducing the efficiency of carrier separation.
5. Interfacial electrode polarization: A capacitive polarization may arise due to the accumulation of charges or ions at interfaces and cause an energy barrier to carrier extraction.

These processes may occur simultaneously, and each process will have a different impact on the hysteresis depending on various parameters such as the device structure, interfacial quality, and the properties of the perovskite layer (grain size, defect density, composition etc.), amongst others.

## 3. Nanostructured Perovskite Absorbers

### 3.1. Introduction

Non-toxic and/or stable materials with similar properties to bulk Pb-OHPs are a high priority and are currently being explored, such as the replacement of Pb with Sn or Bi [23,77], lead-free halide double perovskites [78], and low-dimensional materials [22]. The efficiencies of these solar cells are often far lower than bulk Pb-OHPs and a large amount of development is still required. Nanostructured perovskites include perovskite quantum dots, nanoparticles, nanosheets, nanorods, and perovskites with nanoscale internal ordering. These materials are often termed low-dimensional perovskites (LDPs) and can generally be envisioned by reducing the bulk perovskite structure to the nanoscale in at least one structural dimension.

**Figure 6:**
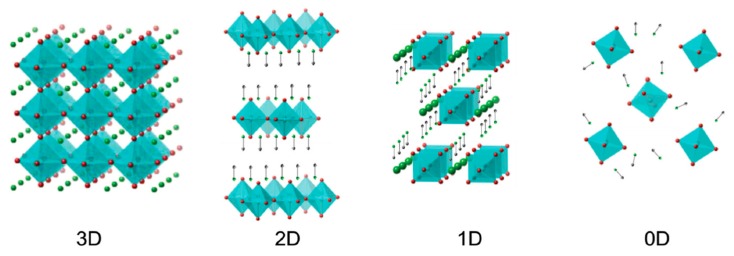
Overview of the different perovskite dimensionalities. Reproduced from ref. [79], with permission from John Wiley and Sons, 2015.

Figure 6 shows schematically how a bulk perovskite with ABX_3_ structure transforms from a three-dimensional perovskite (3DP) to an LDP. In 3DPs, i.e., the typical bulk perovskites used in record-efficiency devices, each BX_6_^4−^ octahedra is connected along all three axes and is anisotropic. It is rather important that this octahedral structure is mostly preserved since the orbital hybridization of B and X sites is responsible for many of the favorable optoelectronic properties of OHPs. For two-dimensional perovskites (2DPs), e.g., nanoplatelets and nanosheets, the BX_6_^4−^ octahedra is connected along two axes and consists of 2D slabs of octahedra with the organic cation occupying the A-site in the voids between slabs. Surrounding the nanosheets are organic ‘barrier’ molecules which prevent the sheets from crystallizing into a larger 3D structure whilst also providing encapsulation and protection against degradation. For one-dimensional perovskites (1DPs), e.g., nanowires and nanorods, the BX_6_^4−^ octahedral network extends along only one axis and is encapsulated with organic barrier molecules. For 1DPs and 2DPs, various organic barriers can be selected, and a wide range of choices exist. Hydrophobic organic barriers can be selected which protect the structure against moisture. For zero-dimensional perovskites (0DPs), the BX_6_^4−^ octahedra is disconnected in all directions and consists of isolated octahedral clusters stabilized by a cationic sublattice. A distinction is often made between 0DPs and quantum dots (QDs), where for a perovskite QD (PQD), the BX_6_^4−^ octahedra remains connected in all three axes and the radius of the particle is below the Bohr exciton radius, whereas for a 0DP each octahedra is completely disconnected from adjacent octahedra, as shown in Figure 6.

Low-dimensional materials can also be produced which are not strictly perovskites yet follow a similar set of design rules; being based on a large heavy metal ion bonded ionically with halide ions, and stabilized by a sublattice of 1+ cations: For example, B-site 3+ cations such as Bi^3+^ form B_2_X_9_^3−^ bioctahedra instead of a BX_6_^4−^ octahedra for 2+ cations, forming the 0DP material (CH_3_NH_3_)_3_Bi_2_I_9_. These materials, which can be produced very similarly to standard perovskites (i.e., from solution) whilst also possessing similar properties, are discussed later. The perovskite term is used loosely to describe these materials, as in some cases the perovskite structure is disturbed.

LDPs exhibit quantum confinement effects which are particularly noticeable through a widening of the bandgap [22]. Although 3DPs already have a bandgap close to the optimum value of ~1.4 eV for a single junction solar cell, a wider bandgap is advantageous for forming tandem devices or for indoor photovoltaics [80]. Furthermore, quantum confinement effects introduce the possibility to reduce losses via carrier multiplication which has already been demonstrated in CsPbI_3_ quantum dots [81] and in the 0DP material (CH_3_NH_3_)_3_Bi_2_I_9_ [82]. The effective use of carrier multiplication in a single-junction solar cell can potentially increase efficiency to ~44% [83], far beyond the Shockley-Queisser (SQ) efficiency limit for a single junction cell of ~33% [84]. In addition, both 3DPs and LDPs are capable of incorporating a low concentration of inorganic nanocrystals into their lattice to form internal energy band alignments which can be used to increase carrier collection and absorption. These hybrid devices can potentially harvest a wide range of the solar spectrum through quantum confinement effects without significantly altering the device architecture, and will be discussed later [85,86].

LDPs often exhibit excitonic behavior as carriers become localized. Since LDPs are often stabilized with organic barriers or a cationic sub-lattice which behaves as an insulating spacer layer, this results in a potential barrier surrounding the individual sheets, rods, or clusters. Carriers therefore become localized on the sheets, rods, or clusters, which often inhibits carrier extraction. The strength of the exciton binding energy is strongly dependent on the dimensionality, with 0DPs usually exhibiting the highest exciton binding energies [87,88].

### 3.2. One- and Two-Dimensional Perovskites

**Figure 7:**
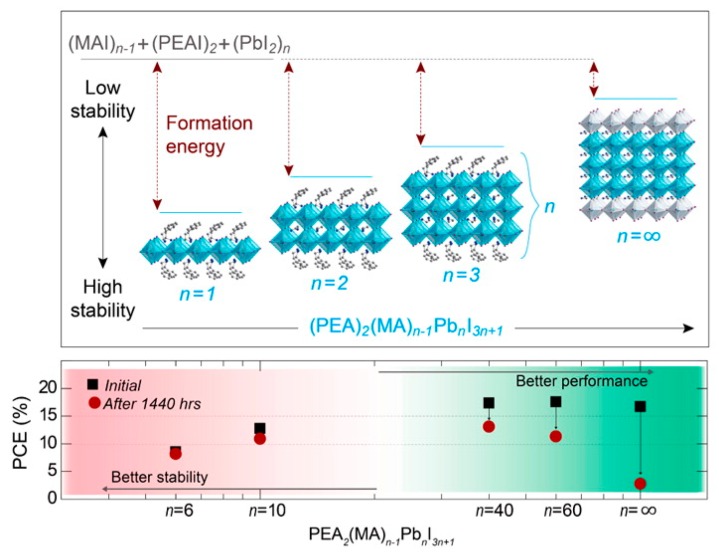
Reducing the dimensionality of organometal halide perovskites leads to higher stability, but lower device performance. Reproduced from ref. [22], with permission from American Chemical Society, 2016

Along with PQDs, perovskite nanosheets and nanorods are the most successful types of LDPs demonstrating the highest efficiencies in photovoltaic devices. The main advantage of reduced dimensionality is that the OHP can be encapsulated with a more stable long chain organic molecule which reduces the rate of degradation. In reference [22] it was shown via simulations that the stability of MAPbI_3_ perovskites can be improved by producing a 2D perovskite encapsulated by larger cations. Further to the benefit of the protective ligands, the 2D perovskite structure has a higher formation energy, which therefore yields a more stable perovskite material. A single 2D slab of the perovskite structure, i.e., a monolayer, encapsulated with organic barrier, is termed *n* = 1, as shown in Figure 7. The bandgap is strongly dependent on the number of perovskite slabs (*n*); as *n* increases, the bandgap narrows and the strength of quantum confinement reduces, and the dimensionality tends towards a quasi-2D structure (*n* > ~10), while for very large values of *n* the perovskite tends towards a 3D structure.

There are a very large number of organic molecules which can potentially be used as the barrier layer, however thus far only a limited number of molecules have been investigated, e.g.: phenylethyl ammonium (C_8_H_9_NH_3_, PEA) [22], benzyl ammonium (C_6_H_5_CH_2_NH_3_, BA) [89], 2-iodoethylammonium (IC_2_H_4_NH_3_) [90], polyethylenimine ((C_2_H_5_N)_n_, PEI) [91], 2-thiophenemethylammonium (C_5_H_7_NS, ThMA) [92], and 3-bromobenzylammonium iodide (BrC_6_H_4_CH_2_NH_2_.HI, 3BBA) [24]. The absorption spectra of 2DPs is weakly associated with the selection of the barrier molecule; optical properties are far more dependent on the *n* value [93]. As *n* tends towards lower values, the stability of the 2DP increases [22], yet the device performance tends to decrease dramatically due to the widening of the band gap and the higher proportion of insulating barrier molecules which have a detrimental effect on carrier transport. Whilst the high in-plane mobility of bulk OHPs is retained along the nanosheets and nanorods, the transport between nanosheets/rods is restricted due to the potential barrier created by the insulating organic barriers which reduces the overall carrier mobility [88]. However, this can be mitigated somewhat by using shorter barrier molecules [94].

**Figure 8:**
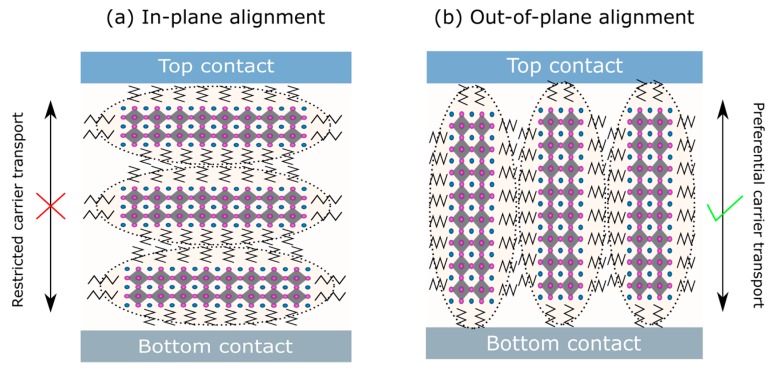
Solar cells based on a perovskite absorber with a two-dimensional network. (**a**) Sheets align parallel with the contacts resulting in low carrier mobility between the contacts and (**b**) sheets align perpendicular to the contacts resulting in favorable out-of-plane mobility between contacts.

In reference [22], the MAPbI_3_ perovskite was reduced to a 2DP and a quasi-2DP structure using PEA barriers with varying *n* values. A quasi-2DP with *n* = 40 was capable of achieving ~15% efficiency, however the stability of quasi-2DPs is still rather poor. Reducing the *n* value to 6 provided high stability, yet the efficiency fell towards ~5%. It is likely that the low efficiency was due to the disordered nature of the sheets which are not aligned perpendicular to the contacts, inhibiting charge transfer. This is shown schematically in Figure 8a. When nanosheets are oriented horizontally, i.e., parallel to the contacts, the charge carrier transfer is restricted in the vertical direction, and charge carrier extraction in inhibited because the long organic barriers separating the LDP sheets inhibit transfer between the layers. 

Higher efficiencies can be achieved by vertically orientating the inorganic sheets, as shown schematically in Figure 8b, whereby charge transport is less restricted. If the nanosheets/rods are orientated vertically, i.e., perpendicular to the contacts (out-of-plane), charge transport is predominantly along the perovskite structure and carrier extraction is therefore far more efficient since carriers must overcome fewer potential barriers. This was initially demonstrated in BA-capped 2DPs with *n* = 3 and the efficiency was increased to over 12% using a hot casting deposition technique to achieve out-of-plane alignment of the 2D sheets [23]. However, these devices still showed rather poor stability when exposed to 65% relative humidity without encapsulation, while fully encapsulated devices demonstrated impressive stability. This has also been demonstrated in perovskite nanorods, with an increase in efficiency from 1.74% to over 15% following out-of-plane alignment [92]. This was achieved by using a methylammonium chloride (MACl) assisted film formation technique which resulted in vertically aligned perovskite nanorods, demonstrating far improved stability over 3D perovskite. Disordered (unaligned) 2DPs usually show significant hysteresis [95], which is likely due to a bias-voltage induced charging effect caused by the insulating organic molecules and poor charge transport when the 2DP sheets are not vertically aligned. However, the hysteresis is mostly eliminated when the nanosheets are aligned out-of-plane with respect to the contacts since charge transport is less restricted [23].

A problem which must be overcome in 2DPs is a stacking misalignment of the 2DP grains which reduces carrier mobility. It was shown that even when 2DPs are aligned with favorable out-of-plane alignment, stacking misalignments between grains restricts charge transfer between vertically aligned sheets [83]. In order to improve device performance, it is important to minimize stacking misalignment between grains. In addition, it was recently demonstrated that it is essential to use LDPs with at least *n* > 2, as it has been shown that exciton dissociation occurs within the nanosheets of 2DPs due to the presence of lower energy states at the edges of the nanosheets which exit only for nanosheets with *n* > 2 [96]. While these edge states are present for 2DPs with *n* > 2, for n ≤ 2, edge-state exciton dissociation was not observed, and the device performance was significantly lower. These lower energy states exist at the edge of 2DPs and provide a favorable energy pathway for excitons to dissociate into free-carriers with longer lifetimes, which was demonstrated to significantly improve device performance. This work demonstrated that it is imperative to synthesize 2DPs with at least *n* = 3 in order to benefit from the favorable exciton dissociation mechanism, even though thinner nanosheets (*n* ≤ 2) can provide higher stability.

**Figure 9:**
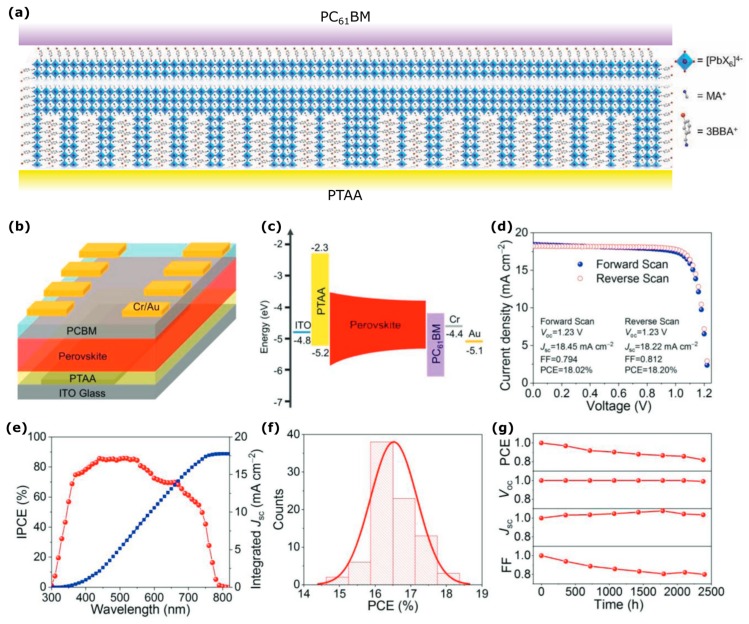
2D perovskite solar cells using 3-bromobenzylammonium iodide barrier molecule. (**a**) Schematic of the device nanostructuring, (**b**) schematic of the device architecture, (**c**) energy band alignment relative to the vacuum level in eV, (**d**) current density-voltage measurement, (**e**) incident photon conversion efficiency (ICPE), (**f**) histogram showing reproducibility of the power conversion efficiency (PCE) and (**g**) solar cell stability for devices stored in the dark between measurements under ≈40 relative humidity. Reproduced with modifications for clarity from ref. [24], with permission from John Wiley and Sons, 2018.

Recent work demonstrated that mixed *n* value 2D perovskites can achieve both favorable carrier transport and band alignment introduced via a unique nanostructuring of the 2D perovskite film, achieving a PCE of 18.2% [24]. The introduction of the barrier molecule 3-bromobenzylammonium iodide (3BBA) leads to the oriented growth of small *n* value 2D perovskites perpendicular to the substrate (*n* ≈ 1–4), followed by the crystallization of large *n* value quasi-2D perovskites in the bulk of the film, shown schematically in Figure 9a and the overall device structure in Figure 9b. This structure also introduces a favorable band alignment as shown in Figure 9c whereby the larger bandgap of the mixed low *n* value 2DPs provides a potential energy gradient driving carriers to the desirable extraction contacts. This demonstrates the remarkable tunability that can be achieved through nanostructuring perovskites to achieve favorable energy band alignment. The devices also showed impressive stability: Unencapsulated devices stored in a dark oven between measurements under ≈40% relative humidity retained 80% of the original PCE after 2400 h. The device could also be submerged underwater for 60 s without any immediate negative effect on the efficiency. It was stipulated that the hydrophobicity due to the presence of iodine in 3BBA results in the enhanced moisture durability of these 2DPs.In general, 2DPs have not been optimized yet via cation engineering to the same extent as 3DPs, which has led to the high performance and improved stability of 3DPs today [13]. Recently, 5% Cs^+^ doping in a 2DP demonstrated an efficiency increase from 12.3% to 13.7%, which was attributed to improved crystal quality and low trap defects, increased grain size, and improved carrier transport [97]. Since most 2DPs with low *n* values show wide bandgaps, it is important to engineer 2DPs which absorb in the visible spectrum. Material engineering and optimization as such demonstrates that there is still great potential for work on improving the 2DPs’ material properties.

Finally, 2DPs may also find use in improving the stability of 3DPs by acting as a protective capping layer. A 2DP was demonstrated as the capping layer in a 3DP solar cell and displayed over 19% efficiency, along with improved stability over the 3DP alone [98]. Further work in this area showed that the deposition of a hydrophobic 2D perovskite on top of a 3D perovskite not only protects against moisture, but also improves carrier extraction. The formation of the 2D perovskite on the surface of the 3D perovskite consumes detrimental and undesirable non-perovskite phases present at the surface of the 3D perovskite and resulted in faster injection of holes into the HTL [99]. More recently, an ammonium salt post-treatment of a 3D OHP film increased the PCE from 20.5% to 22.3% via the formation of a 1DP passivation layer [100]. Devices retained 95% of the initial PCE after continuous illumination for 550 h. This area of work presents a route towards avoiding the necessity for encapsulants in PSCs, therefore reducing costs and avoiding issues pertaining to thermal expansion mismatch.

### 3.3. Zero-Dimensional Perovskites

Pb-based 0DPs have been previously studied but so far seem unsuitable for photovoltaics [101,102]. For example, when the typical perovskite MAPbI_3_ is transformed into a 0DP with the chemical formula (CH_3_NH_3_)_4_PbI_6_, the structure is extremely unstable [101]. Alternatively, more stable inorganic Pb-based 0DPs can be produced such as Cs_4_PbBr_6_, however, the bandgap is very large: Pb- based 0DPs tend to have very large bandgaps which are unsuitable for photovoltaics, typically in the UV-range, irrespective of the halide anion selected [102]. 

**Figure 10:**
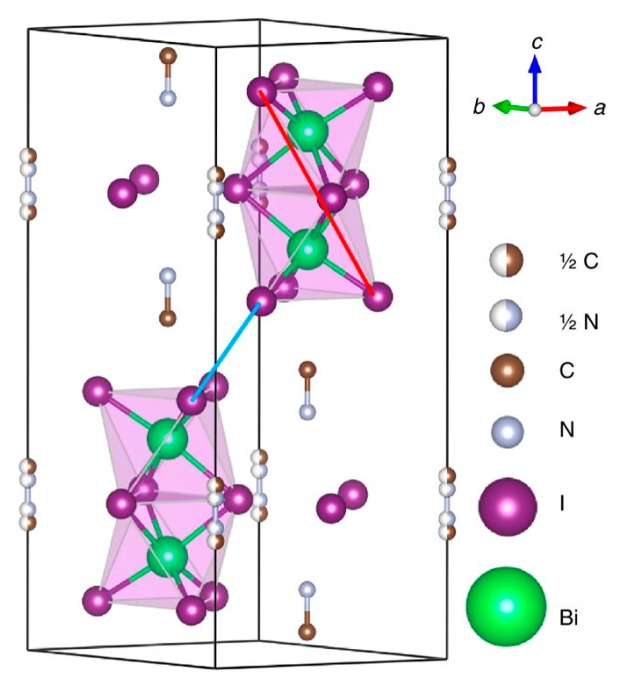
Schematic of the structure of (CH_3_NH_3_)_3_Bi_2_I_9_ which forms a zero-dimensional network. Bi_2_I_9_^3-^ clusters are stabilized within a (CH_3_NH_3_)^+^ ionic lattice. Reproduced from ref. [82], with permission from Springer Nature, 2017.

Alternatively, Bi-based 0DPs have bandgaps closer to 2 eV and have been demonstrated as the absorber in photovoltaic cells [86,103,104,105]. Bi, which is adjacent to Pb in the periodic table, has a similar atomic radius to Pb yet with one additional valence electron yielding 3+ instead of 2+, resulting in a B_2_X_9_^3−^ bioctahedral structure rather than the BX_6_^4−^ octahedral structure. These perovskite structures have the formula A_3_B_2_X_9_, but can also be expressed as AB_2/3_X_3_, i.e., a metal-deficient perovskite. Figure 10 shows the structure of a 0DP with the chemical formula (CH_3_NH_3_)_3_Bi_2_I_9_, where Bi_2_I_9_^3−^ clusters are separated by a CH_3_NH_3_^+^ cationic lattice. Here, CH_3_NH_3_^+^ can be replaced with a range of organic and inorganic cations. Whilst these materials are often referred to as ‘perovskites’, their crystallographic structure is slightly different to the perovskite structure, whereby the BX_6_^4-^ octahedra is instead replaced with a B_2_X_9_^3−^ bioctahedra.

Bi-0DPs have been studied and the best devices have achieved efficiencies of 1.64% [105]. These materials, with bandgaps of ~2 eV, generally exhibit high exciton binding energies (~300 meV) and high effective masses for carriers [88]. Because of the excitonic nature of these materials with quantum confinement effects, 0DPs have been shown to exhibit carrier multiplication [82]. However, due to the high exciton binding energy, the rates of electron-hole recombination is high which limits device performance. 0DPs also exhibit anisotropic carrier mobilities if the cluster is non-symmetrical and/or if the spacing between clusters varies between planes [88]. It is therefore necessary to try to overcome the high exciton binding energy and carrier transport issues by a range of possible methods, such as modifying the cationic sub-lattice, using semiconducting polymers which enhance carrier mobility between the clusters, or by forming hybrids with inorganic nanocrystals which assist in exciton dissociation.

(CH_3_NH_3_)_3_Bi_2_I_9_ solar cells can be processed and stored entirely in ambient conditions and have demonstrated far superior stability to 3DPs, likely due to the formation of a native surface layer of Bi_2_O_3_/BiOI which provides self-encapsulation of the perovskite [30]. This layer does not inhibit carrier extraction, and is also likely responsible for the negligible hysteresis observed in these devices [86]. If 0DPs were employed as the wide-bandgap top cell in a tandem solar cell, their high stability can provide encapsulation for the less-stable OHP bottom cell to prevent moisture ingress. Furthermore, the absorption can be modified by incorporating optically active organic molecules or forming hybrids with nanocrystals with suitable band alignment [86], and the large bandgap of 2 eV can be reduced to values as low as 1.45 eV through doping and/or changing the A-site cation [106,107,108].

Sb-based 0DPs have also been demonstrated with the formula (CH_3_NH_3_)_3_Sb_2_I_9_ and have so far achieved higher efficiencies than Bi-0DPs, with the best devices so far achieving 2.77% efficiency [109]. The higher efficiencies of these devices is likely due to the intrinsically lower exciton binding energy of Sb-0DPs [110]. Since the bandgap of Sb-0DPs is still quite large (~1.9 eV), researchers have attempted to lower the bandgap through Sn-doping, and successfully reduced the bandgap to 1.53 eV with 40% replacement of Sb with Sn to form (CH_3_NH_3_)_3_Sb_0.6_Sn_0.4_I_9_. Doping with Sn increased the efficiency of the devices from 0.57% (without Sn, bandgap = 2.0 eV) to 2.7% (40% Sn, bandgap = 1.53 eV). Since the starting efficiency of the undoped Sb-0DP reference device was quite low (0.57%) compared to the highest reported in the literature (~2.77%), it is likely that through device optimization of the Sn-doped Sb-0DP will quickly lead to higher efficiencies in the near future, likely exceeding 5%. These Sn-doped Sb-0DPs demonstrated impressive stability with no change in the XRD spectra after 15 days of exposure to ambient conditions. Although inorganic 0DPs have also been produced with the formula Cs_3_Sb_2_I_9_ and Cs_3_Bi_2_I_9_, these devices tend to show very low efficiencies below 0.1% [111,112], likely due to their large bandgaps and high exciton binding energy, and have therefore not been pursued to the same extent.

### 3.4. Perovskite Quantum Dot Solar Cells

High exciton binding energies and inefficient charge transfer are significant issues associated with LDPs which limit carrier extraction, therefore inhibiting device performance. This can potentially be overcome in PQDs through close-packing with electronic coupling between QDs. Colloidal PQDs can be readily synthesized from solution using organic capping molecules, such as oleic acid, oleylamine, octadecene, etc. which prevent the perovskite from forming into a larger crystal [113]. These long chain molecules must be removed during device fabrication for efficient solar cell performance. However, PQDs with organic A-site cations are often highly unstable, and it is therefore not possible to remove these barrier molecules as they are essential for preventing rapid degradation. As discussed previously, the issue of long chain organic barrier molecules in perovskite nanorods and nanosheets can be overcome by aligning the sheets and rods perpendicular to the contacts, minimizing the number of potential barriers that must be overcome by charge carriers. However, due to the spherical shape of QDs, this type of favorable alignment is not possible, and researchers must therefore look towards inorganic PQDs which do not require encapsulation in protective organic barriers or use a different architecture [85,114].

All-inorganic perovskites can be formed by replacing the A-site with an inorganic cation, such as Cs^+^, e.g., CsPbI_3_. Inorganic PQDs such as CsPbI_3_ are the most favorable perovskite material since the bandgap of bulk CsPbI_3_ is the smallest of the inorganic perovskites (1.73 eV for the cubic phase) [115]. However, accessing the desired cubic phase of CsPbI_3_ is challenging: For bulk CsPbI_3_, the orthorhombic phase is thermodynamically preferred at room temperature, but the large bandgap of 2.82 eV renders orthorhombic CsPbI_3_ unsuitable for photovoltaics [115]. The cubic phase exhibits a more favorable bandgap of 1.73 eV; however, this phase is unstable at room temperature. Forming CsPbI_3_ quantum dots enabled researchers to achieve the cubic phase at room temperature, as the contribution of the surface energy for CsPbI_3_ quantum dots was shown to retain the favorable cubic perovskite phase [114]. 

CsPbI_3_ PQD solar cells were fabricated with 10.77% efficiency [114]. These devices could be fabricated at ambient conditions and showed impressive stability when stored in a desiccator, with no decrease in performance after 60 days. However, when stored in relative humidity of 40–60% there was a significant decrease in the device performance after just 2 days, although QD devices demonstrated improved stability over bulk CsPbI_3_. Furthermore, CsPbI_3_ QD devices showed significant hysteresis, likely due to difficulties associated with charge transfer between quantum dots, ion migration, and charge trapping at QD surfaces. 

**Figure 11:**
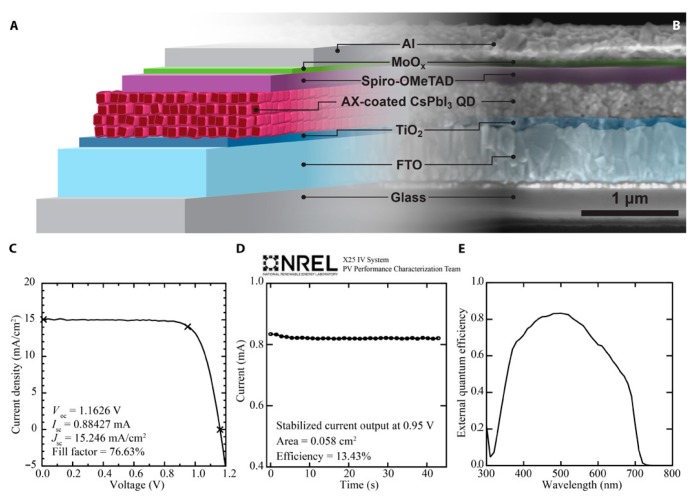
CsPbI_3_ quantum dot solar cells. (**A**) Schematic of the device structure, (**B**) cross-sectional scanning electron microscopy image, (**C**) current density-voltage scans under solar simulated light, (**D**) stabilized current at a constant voltage of 0.95 V, and (**E**) external quantum efficiency. Reproduced from ref. [21], with permission from AAAS, 2017.

These devices were later improved by a post treatment of the CsPbI_3_ QDs, and increased the efficiency to 13.43%, as shown in Figure 11 [21]. This was achieved through efficient QD coupling via a post-treatment of the film, allowing improved change transfer between the QDs in the film. The post-treatment involved soaking the CsPbI_3_ QD thin film in a formamidinium iodide in ethyl acetate solution for 10 s. The post-treatment creates a coating on the CsPbI_3_ QDs and does not alter their nanocrystalline character. It was confirmed that the post-treatment improved the carrier mobility from 0.23 to 0.50 cm^2^ V^−1^ s^−1^. However, the poor stability of CsPbI_3_ at ambient conditions has not yet been addressed, and it is likely that these materials will require encapsulation. Alternatively, a Cs- salt post-treatment was reported achieving PCE of 14.1% [116]. The Cs-salt treatment is performed after the removal of ligands from the CsPbI_3_ QDs. When the ligands are removed, Cs vacancies are left behind on the CsPbI_3_ QDs. These vacancies are filled by Cs via a Cs-salt post treatment, resulting in improved free carrier mobility, lifetime, and diffusion length, as well as greater stability over untreated CsPbI_3_ QDs.

One of the advantages of CsPbI_3_ QDs is the possibility of carrier multiplication, which has already been demonstrated in CsPbI_3_ QDs with a high carrier multiplication quantum yield of 98% [81]. While the bandgap for quantum confined materials scales as *E*_g_ ~ 1r where *r* is the radius, the rate of Auger recombination scales as 1r6 and therefore forming smaller QDs is more favorable for carrier multiplication. The average radius of the QDs in this work was 5.75 nm and the exciton Bohr radius for CsPbI_3_ QDs is 6 nm. The QDs are therefore in the weak quantum confinement regime, yet still exhibited highly efficient carrier multiplication indicating that strong quantum confinement is not necessary in these materials for carrier multiplication [81].

**Figure 12:**
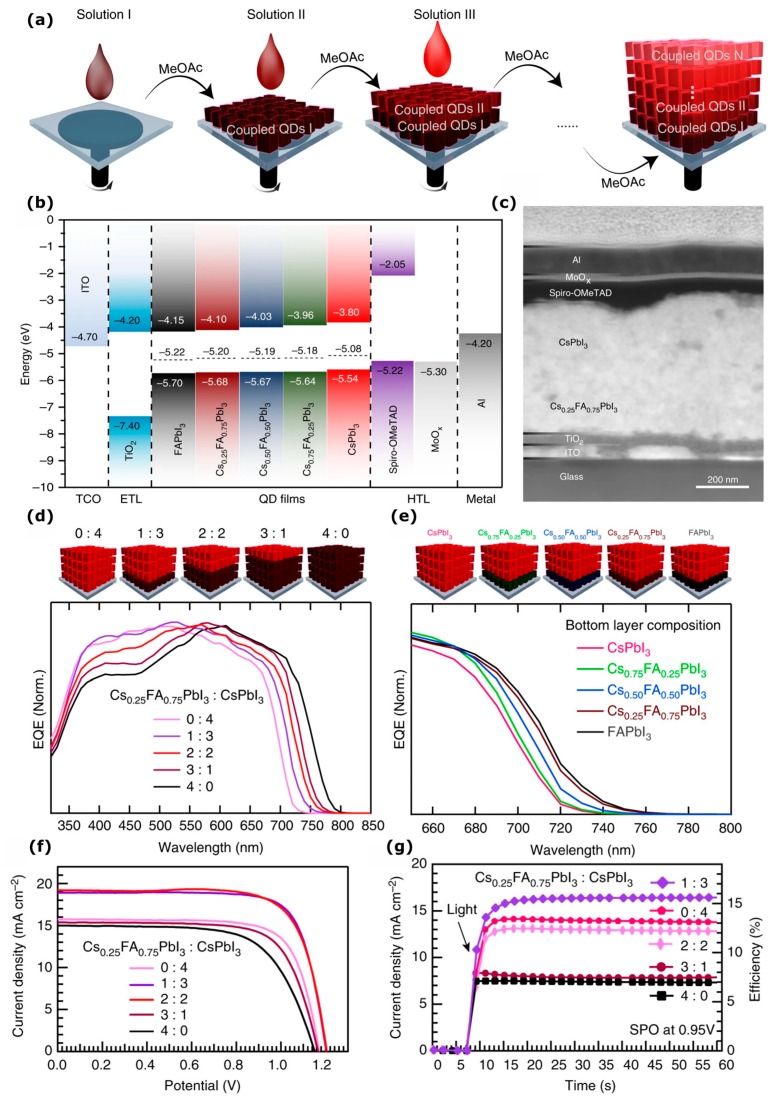
Perovskite quantum dot (PQD) solar cells with charge separating heterostructure. (**a**) Schematic of the device fabrication via spin coating, (**b**) energy band structure of the various PQDs used in the study, (**c**) cross-sectional scanning electron microscope of a typical device, (**d**) the external quantum efficiency (EQE) of solar cells made with various ratios of Cs_0.25_Fa_0.75_PbI_3_ to CsPbI_3_ quantum dots, (**e**) EQE at the absorption edge of various quantum dots in the series Cs_x_FA_1-x_PbI_3_ as the bottom layer. (**f**) current density-voltage (JV) curves for the devices shown in (**d**) and (**g**) stabilized power output (SPO) of the varying compositions shown in (**f**). Reproduced from ref. [117], with permission from Springer Nature, 2019.

The band energy structure of the active layer can be tuned to achieve improved carrier extraction by using PQDs with varying condition band, valence band, and Fermi level positions. The sequential deposition of PQDs with varying band energy positions has been shown to improve carrier extraction [117] and is reproduced in Figure 12. A schematic of the sequential deposition of PQDs is shown in Figure 12a and the band energy positions of the PQDs studied in this work are shown in Figure 12b. PQDs were synthesized in the series Cs_x_FA_1-x_PbI_3_ and PQD heterojunction devices were fabricated with the structure ITO/TiO_2_/PQDs I/PQDs II/spiro-MeOTAD/MoO_x_/Al. The best device performance was obtained using either Cs_0.5_FA_0.5_PbI_3_ or Cs_0.25_FA_0.75_PbI_3_ as the bottom layer and CsPbI_3_ on the top. Devices based on a Cs_0.25_FA_0.75_PbI_3_:CsPbI_3_ heterojunction were investigated further for optimization. Figure 12c shows the SEM cross section of the device and Figure 12d shows the effect of varying the thickness ratio of Cs_0.25_FA_0.75_PbI_3_:CsPbI_3_ on the EQE spectra. A ratio of 1:3 (Cs_0.25_FA_0.75_PbI_3_:CsPbI_3_) retained most of the short wavelength EQE contribution from CsPbI_3_ whilst also red-shifting the EQE onset slightly. Higher proportions of Cs_0.25_FA_0.75_PbI_3_ lead to a fall in EQE at shorter wavelengths, despite red-shifting the EQE onset more. Figure 12e shows that varying the bottom layer composition, i.e., by fabricating devices with the structure ITO/TiO_2_/Cs_x_FA_1-x_PbI_3_/CsPbI_3_/spiro-MeOTAD/MoO_x_/Al for x = 0.25, 0.5 and 0.75 leads to a similar red-shift in the EQE as the bandgap of the Cs_x_FA_1-x_PbI_3_ PQDs is decreased. The J-V characteristics are shown in Figure 12f, and ratios of 1:3 and 2:2 achieve the highest PCEs, however due to the large hysteresis present in these devices, the SPO was also presented and revealed that devices with a 1:3 ratio of Cs_0.25_FA_0.75_PbI_3_:CsPbI_3_ achieved the highest SPO at 15.52%. Finally, bulk heterojunction architecture devices were also fabricated by mixing the PQDs. These devices did not exhibit the same enhanced performance confirming that a bi-layer heterojunction of PQDs is essential for achieving improved carrier collection.

A summary has been provided in Table 1 comparing a selection of the most notable results since 2018 for 0D, 1D, 2D and QD perovskites, as well as also including some of the notable heterojunctions formed between 3D perovskites and LDPs. This table also provides a summary of the stability of the solar cell devices, noting the storage conditions and the solar cell J-V measurement type (i.e. continuous or intermittent, where continuous measurements typically involve the device remaining under constant solar simulated light, whilst for intermittent measurements the device is removed from illumination and stored in specified storage conditions between measurements).

### 3.5. Perovskite-Nanocrystal Hybrid Devices

The formation of hybrid layers and devices through incorporating nanocrystals into the OHP layer have also been explored, both in bulk 3DPs [85] and in 0DPs [86]. The introduction of quantum-confined NCs to bulk 3D OHPs enables the possibility of carrier multiplication. Thus far, SiNCs have been primarily studied in this context, since most nanocrystals studied for organic-inorganic hybrid photovoltaics are toxic Pb- or Cd-based [20], and it would be counterintuitive to add a toxic material to a lead-free perovskite. SiNCs are an environmentally-friendly material which are non-toxic and can be synthesized through a wide variety of methods [124,125]. The properties of SiNCs can also be easily modified by surface engineering and the absorption and emission properties can be influenced by the surface terminations [125,126,127]. Surface engineering can also improve carrier transport in SiNCs by passivating surface defects [128]. While SiNCs do present their own challenges, they represent an important model NC material.

It was previously demonstrated that the incorporation of silicon nanocrystals (SiNCs) into the 0DP with the formula (CH_3_NH_3_)_3_Bi_2_I_9_ led to an enhancement in the device performance [86]. It was proposed that the SiNCs may act as a dissociation pathway for tightly-bound excitons on the nanoclusters of Bi_2_I_9_^3-^ bioctahedra. An electronic junction formed between the perovskite material and the inorganic nanocrystal can provide an energetically favorable pathway for excitons to overcome the potential barrier created by the cationic sublattice, providing exciton dissociation before the carrier recombines. Once the exciton is dissociated it becomes a free-carrier which can be extracted. This is commonly employed in organic–inorganic hybrid solar cells using SiNCs to enhance exciton dissociation [129]. These types of hybrids may present a route towards significantly improving the efficiency of LDPs.

**Figure 13:**
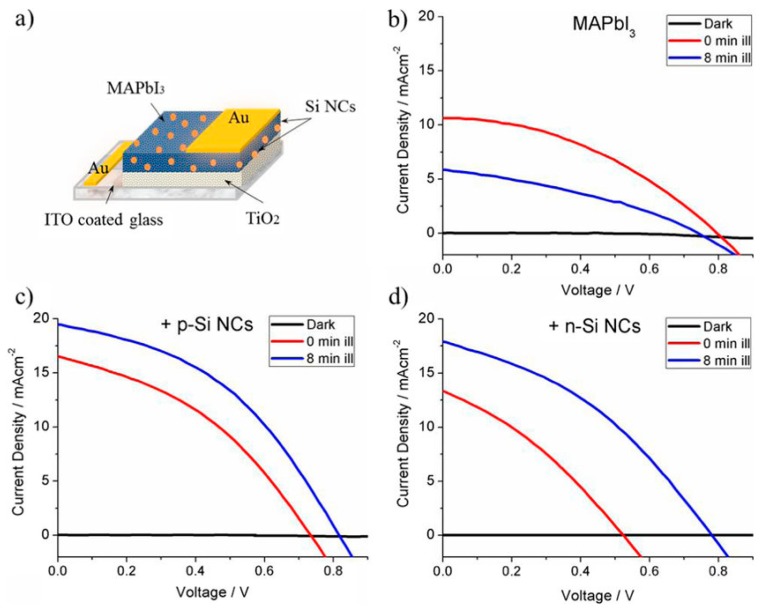
Perovskite-silicon nanocrystal (SiNC) hybrid solar cells show improved device performance especially after light-soaking. (**a**) Schematic of device structure, and current-density voltage (JV) curves for (**b**) MAPbI_3_ alone, (**c**) MAPbI_3_ with p-type SiNCs, and (**d**) n-type SiNCs. Reported from ref. [85], with permission from Elsevier, 2018.

Hybrid MAPbI_3_-SiNC devices also exhibit improved device performance and stability [85]. X-ray photoelectron spectroscopy (XPS) indicated that MAPbI_3_ bonds with SiNCs via intermediate oxide bonds with nitrogen in methylammonium (N–O–Si). The oxidation of SiNCs was also observed in XPS and is likely responsible for the improved stability, whereby SiNCs may act as a ‘sponge’ absorbing oxidizing species in the MAPbI_3_ layer resulting in slight oxidation of the SiNCs. Furthermore, hybrid devices with SiNCs exhibited improved device performance after light soaking for 8 min (Figure 13), whilst the performance of MAPbI_3_-only devices decreased. This is commonly observed in MAPbI_3_ devices and is attributed to light-activated trap states with inhibited photocarrier extraction [130]. The observation of the inverse behavior in hybrid devices suggests that SiNCs may inhibit defect migration possibly via bonding with the perovskite structure.

**Figure 14:**
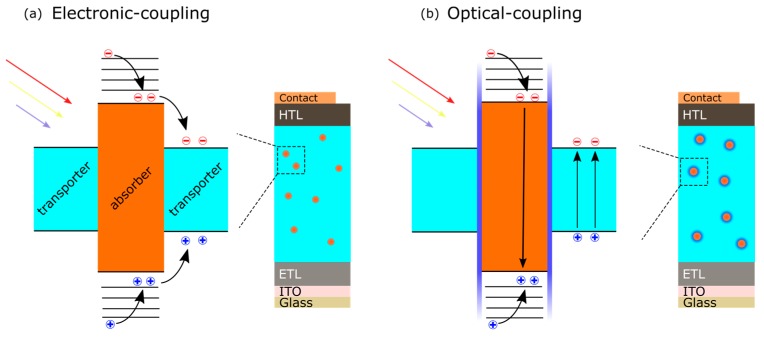
Inverted type-I band alignment: (**a**) electronically coupled and (**b**) optically coupled. Reproduced from ref. [85], with permission from Elsevier, 2018..

In addition, incorporating nanocrystals into OHPs presents the opportunity to create various types of favorable band alignment between the OHP and the nanocrystal. Coupling the properties of nanocrystals with perovskites can lead to improvements in device performance and opens up an avenue of possibilities to exceed the SQ-limit. Forming an inverted type-I junction can potentially improve carrier collection either through optical coupling or electronic coupling. MAPbI_3_-SiNC hybrid devices form an inverted type-I band alignment (Figure 14), where wider-bandgap SiNCs were incorporated into the perovskite layer with electronic and/or optical coupling with the OHP, depending on whether or not the SiNCs are oxidized [85]. In an electronically coupled inverted type- I junction, the absorption in the wider-bandgap nanocrystal generates carriers which can be transferred into the adjacent conduction and valence bands of the smaller bandgap perovskite. In an optically coupled system, the nanocrystal behaves as a ‘interpenetrated’ down converter for high energy photons, where radiative carrier recombination via photoluminescence (PL) results in excitations in the narrow bandgap perovskite. It is therefore important that the peak PL emission is tailored to the bandgap of the perovskite to maximize the conversion efficiency. In MAPbI_3_:SiNC hybrid devices, it is expected that the structure initially forms an electronically coupled junction whereby carriers generated in the SiNCs can transfer into the OHP. After oxidation, carriers generated in SiNCs are trapped by the oxide potential barrier and recombine via photoluminescence, thus generating an optically coupled junction. It was found using Kelvin probe and XPS that the type-I band alignment is preserved even after the SiNCs became oxidized [85]. These new architectures represent new opportunities for exploring different combinations of materials with perovskite structures.

### 3.6. Perovskite Oxide Nanoparticles

Perovskite oxides (ABO_3_) are attractive materials for photovoltaics because of the possibility of low-cost, non-toxic photovoltaics with high stability [131]. However, most semiconducting perovskite oxides have large bandgaps (~3–5 eV) due to oxygen-metal transitions with large differences in their electronegativities [132,133], and are therefore generally unsuitable for absorbing light within the solar spectral range. Attempts to reduce the bandgap of perovskite oxides include doping [134], intrinsic defects [135], forming oxynitrides [136], solid solutions [137], and cationic ordering [132].

Perovskite oxides and their derivatives (layered perovskite oxides) represent a large family of materials which exhibit a multitude of properties, and have been investigated for applications including photovoltaics [138]. Perovskite oxides possess a high-degree of flexibility given that 90% of the metallic natural elements in the periodic table can adopt a stable perovskite-type oxide structure [139]. There remains a significant opportunity for exploring the use of metal oxides in photovoltaics to achieve affordable solar cell devices with high efficiency and tunability, whilst easily meeting the often elusive requirement of high stability. The use of metal oxides with highly-tunable absorption properties via the introduction of vacancies [135] and doping [140] would allow for the facile fabrication of multi-junction devices with high stability.

Ferroelectric perovskite oxides have been demonstrated in photovoltaics [133], however they tend to possess large bandgaps (~3–5 eV) and low conductivities, and therefore efficiencies are low (~1%). Plasmonic perovskite oxides have not been explored to the same extent for photovoltaics. Perovskite oxides can be heavily doped to be plasmonic or can be achieved through structural vacancies to strongly modify electronic properties [140]. However, one of the issues associated with plasmonic materials is carrier extraction, and therefore forming extremely thin absorber layers using nano-sized plasmonic oxides is necessary to rapidly extract carriers before recombining.

**Figure 15:**
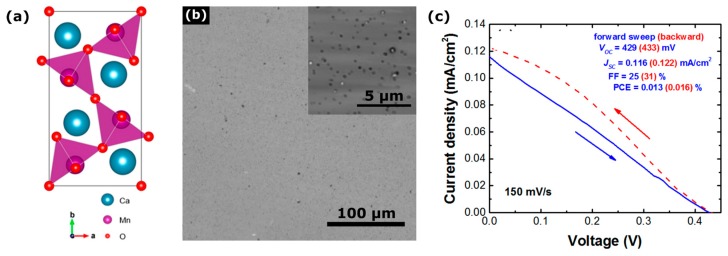
(**a**) Structure of CaMnO_2.5_ reproduced from ref. [141], with permission from American Chemical Society, 2014, (**b**) optical microscope images of CaMnO_2.5_ after laser fragmentation, the inset shows a high-magnification optical microscope image, and (**c**) current density-voltage characteristic of a CaMnO_2.5_ solar cell under solar simulated light.

The perovskite oxide CaMnO_3_ is an orthorhombic perovskite, and upon reduction in flowing Ar gas the structure can be transformed to an oxygen-deficient perovskite with the structure CaMnO_2.5_. The structure of CaMnO_2.5_ is essentially an orthorhombic perovskite with an internal 1D nanostructure ordering as shown in Figure 15a. The introduction of oxygen vacancies removes one oxygen atom from each MnO_6_ octahedra and results in a square pyramid of MnO_5_. This structural transformation reveals many interesting properties, such as plasmonic behavior and significantly improved electrocatayltic and photocatalytic activity [141]. CaMnO_2.5_ can be described as an orthorhombic perovskite because the Ca and Mn perovskite sub-lattice is preserved. Since the resulting powder is phase pure, the oxygen vacancies are expected to be ordered resulting in 1D chains of MnO_5_ square pyramids [141]. The MnO_5_ square pyramids are connected along a one- dimensional network extending through the crystal connected by oxygen atoms, which may be favorable for carrier extraction. This oxygen deficiency creates an internal molecular level porosity. The one-dimensional network of MnO_5_ pyramids may also enhance charge transport and enable efficient carrier separation whereby photoexcited carriers are transported along segregated Mn–O carrier transport channels. CaMnO_2.5_ displays broad absorption of light from the infra-red through to the visible region of the solar spectrum. Nanoparticles of CaMnO_2.5_ can be easily produced via a sol-gel process followed by reductive annealing, and then deposited as an ultrathin film either by spray coating or spin coating. The shape and size of the CaMnO_2.5_ nanoparticles is shown in Figure 15b. CaMnO_2.5_ nanoparticles have been successfully used to fabricate a photovoltaic cell, and the device performance is shown in Figure 15c. While initial device performance was low, this work serves as a proof of concept and it is likely that the efficiency can be significantly improved, primarily through optimization of the layer thickness and interfacial engineering to improve coupling between CaMnO_2.5_ nanoparticles and transport layers.

## 4. Conclusions and Outlook

This review article has provided a summary of 3D bulk OHPs and an overview of the recent direction and progress towards LDPs. To date, 1DPs and 2DPs have shown the highest efficiencies, yet it is unclear whether these materials will suffer the same long-term stability issues as bulk OHPs. Nanosheets with *n* ≤ 2 tend to show impressive stabilities but suffer from low performance issues, particularly due to their very large bandgaps. It is still unclear whether 2DPs and 1DPs with *n* > 2 can demonstrate the long-term stability required for commercialization. Furthermore, the issue of stacking faults between grains, which inhibits charge transfer through the layer, must be overcome to increase the efficiency towards 20%. Exploring conductive organic barriers could be a possible route towards overcoming carrier transport issues.

0DPs tend to be highly stable, however their efficiencies are often very low due to issues associated with carrier extraction, where excitons tend to be strongly localized on BX_6_^4−^ or B_2_X_9_^3−^ clusters. Methods to enhance exciton dissociation and carrier transport need to be further explored if these materials are to demonstrate noteworthy efficiencies in photovoltaics, particularly through forming hybrids with nanocrystals to promote exciton dissociation, and by exploring various ion substitutions at the A-site to lower the exciton binding energy. Provided that these challenges can be overcome, 0DPs with large bandgaps can be incorporated as a top cell in a tandem solar cell. For use in single junction cells, it is important to explore doping along with varying the A-site ion with the aim of discovering 0DPs with smaller bandgaps, which are currently often >2 eV.

PQDs have shown impressive performance so far, yet the choice of materials is rather limited due to the poor stability of organometal PQDs, which are unstable unless capped with long chain organic barriers which inhibit carrier transport. Inorganic CsPbI_3_ QDs do not require capping molecules and have demonstrated improved short-term stability along with impressive solar cell efficiencies over 13%. Despite this, CsPbI_3_ QDs are highly unstable in ambient conditions and encapsulation of the entire solar cell device is essential. As research in this field is still in its infancy, there are limited studies on the stability of CsPbI_3_ QDs and the extent to which the stability can be improved remains unclear. It is therefore currently difficult to predict the potential for CsPbI_3_ QDs in photovoltaics.

Hybrid devices can be formed by adding NCs to bulk 3DPs or 0DPs. These hybrid device architectures have been explored using SiNCs, demonstrating an improvement in the device performance due to the possibility of a type-I band alignment which can be optically and/or electronically coupled to improve carrier collection. Furthermore, adding SiNCs indicates a route towards extending the device lifetime, whereby SiNCs are oxidized by the residual moisture in the layer rather than degrading the OHP. This was shown to preserve the favorable type-I band alignment without affecting the device performance.

Due to the significant stability issues suffered by OHPs, occurring both in bulk and low- dimensional forms, we have also briefly introduced the field of perovskite oxide nanomaterials, studying the oxygen-deficient perovskite CaMnO_2.5_. This material, which absorbs a broad range of light in the solar spectrum from infrared to ultra-violet, has a one-dimensional internal structure which may promote carrier transport. Although the efficiency of the solar cell device is low, there remains significant opportunities for tuning the properties, optimizing devices, and exploring doping to improve device performance.

Finally, while the efficiencies of LDPs are still often far lower than bulk-OHPs, it is encouraging that higher device efficiencies are continually being reported. Provided that these devices can be fabricated with efficiencies of >20%, it is likely that they will be attractive to the market assuming they can be produced at very low cost and with far superior stability to 3D OHPs.

## Figures and Tables

**Table 1:** A selection of notable reports on low-dimensional perovskite solar cells. QDs, PCE, RT, and RH stand for quantum dots, power conversion efficiency, room temperature, relative humidity, respectively.

Dimensionality	Material (*n* Value) ^1^	PCE (%)	Stability	Reference (Year)
0D	(CH_3_NH_3_)_3_Sb_2_I_9_	2.77	Retained 80% of initial PCE after 3 h under constant illumination, ambient conditions, encapsulated.	[109] (2018)
0D	Cs_3_Sb_2_I_9_	1.21	Retained 95% of initial PCE after 60 days, intermittent measurements, stored at RT, 50% RH, unencapsulated.	[118] (2019)
0D	Cs_3_Sb_2_I_9_	1.49	Retained >80% of initial PCE after 30 days, intermittent measurements, storage conditions unspecified.	[119] (2018)
1D	(ThMA)_2_(MA)_n−1_Pb_n_I_3n+1_ (*n* = 3)	15.42	Retained 90% of initial PCE after 100 h, intermittent, stored in N_2_ in the dark, unencapsulated.	[92] (2018)
1D, mixed with 3D MAPbI_3_	1,4-benzene diammonium (BDA)-PbI_4_ (*n* = 1)	14.1	Retained 95% of original PCE after >1000 h, intermittent measurement, stored in dark at RT, 85% RH, encapsulated.	[120] (2019)
1D/3D heterostructure	ethylammonium iodide (EAI)-treated FA_0.93_Cs_0.07_PbI_3_	22.3	Retained 95% of initial PCE after 550 h, continuous measurement under constant illumination in N_2_ atmosphere at RT, unencapsulated.	[100] (2019)
2D	(FPEA)_2_MA_4_Pb_5_I_16_ (*n* = 5)	13.64	Retained 65% of initial PCE after 576 h, ambient air at 70 °C unencapsulated.	[121] (2019)
2D	(BzDA)A_9_Pb_10_ (I_0.93_Br_0.07_)_31_ (*n* = 10)	15.6	Retained 80% of initial PCE after 84 h, intermittent measurements, kept in dark at RT in ambient air, RH = 20–50% unencapsulated.	[122] (2019)
Quasi-2D	3-bromobenzylammonium iodide (BBAI)- (*n* = 2)	18.2	Retained 82% of initial PCE after 2400 h, intermittent measurements, stored in dark at RT, ~40% RH, unencapsulated.	[24] (2018)
Quasi-2D	(BE)_2_ (FA)_8_Pb_9_I_28_ (*n* value not reported)	17.4	Retained 80% of initial PCE after 50 h, stored in the dark at RT, RH = 80%, unencapsulated.	[123] (2018)
QDs	CsPbI_3_	14.1	Retained 70% of initial PCE after 50 h, intermittent measurements, stored in the dark at RT and 40% RH, unencapsulated.	[116] (2019)
QDs	CsPbI_3_:Cs_0.25_FA_0.75_PbI_3_	17.4	Retained 80% of initial PCE after 10 h, intermittent measurements under constant illumination at 40 °C and 25% RH, encapsulated.	[117] (2019)

^1^ *n* values are only applicable for 1D and 2D materials.
